# Liquid–liquid phase separation of microtubule‐binding proteins in the regulation of spindle assembly

**DOI:** 10.1111/cpr.13649

**Published:** 2024-05-13

**Authors:** Shuang Sun, Yang Yang, Jun Zhou, Peiwei Liu

**Affiliations:** ^1^ Center for Cell Structure and Function, Shandong Provincial Key Laboratory of Animal Resistance Biology, Collaborative Innovation Center of Cell Biology in Universities of Shandong, Institute of Biomedical Sciences, College of Life Sciences Shandong Normal University Jinan China; ^2^ Translational Medicine Center The First Affiliated Hospital of Zhengzhou University Zhengzhou China; ^3^ State Key Laboratory of Medicinal Chemical Biology, Haihe Laboratory of Cell Ecosystem, College of Life Sciences Nankai University Tianjin China

## Abstract

Cell division is a highly regulated process essential for the accurate segregation of chromosomes. Central to this process is the assembly of a bipolar mitotic spindle, a highly dynamic microtubule (MT)‐based structure responsible for chromosome movement. The nucleation and dynamics of MTs are intricately regulated by MT‐binding proteins. Over the recent years, various MT‐binding proteins have been reported to undergo liquid–liquid phase separation, forming either single‐ or multi‐component condensates on MTs. Herein, we provide a comprehensive summary of the phase separation characteristics of these proteins. We underscore their critical roles in MT nucleation, spindle assembly and kinetochore‐MT attachment during the cell division process. Furthermore, we discuss the current challenges and various remaining unsolved problems, highlights the ongoing research efforts aimed at a deeper understanding of the role of the phase separation process during spindle assembly and orientation. Our review aims to contribute to the collective knowledge in this area and stimulate further investigations that will enhance our comprehension of the intricate mechanisms governing cell division.

## Graphical abstract

The nucleation and dynamics of microtubules (MTs) are intricately regulated by MT‐binding proteins. Over the recent years, various MT‐binding proteins have been reported to undergo liquid–liquid phase separation, forming either single‐ or multi‐component condensates on MTs. Herein, we summarise the phase separation characteristics of these proteins.

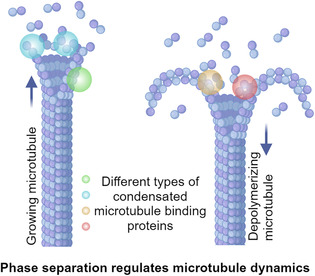

## INTRODUCTION

The precise segregation of chromosomes during mitosis and meiosis, orchestrated by the spindle, is vital for cell proliferation and development. The initiation of bipolar spindle, the microtubule (MT) based superstructure, occurs through the separation of duplicated centrosomes before the nuclear envelope breakdown in prophase. Simultaneously, the centrosomes form the spindle poles.^1^, ^2^ Following the nuclear envelope breakdown, MTs from the two spindle poles attach to chromosomes at kinetochores, interacting with each other throughout the prometaphase and metaphase.^3^, ^4^ Astral MTs engage with the cell cortex through interactions with cortical factors. MTs, along with associated binding proteins, collectively generate the forces necessary for the precise movement and segregation of chromosomes during metaphase and anaphase.^5^, ^6^ After chromosome segregation in anaphase, the mitotic spindle disassembles, and the central spindle assembles at the cell equator, facilitating cytokinesis in telophase.

Most cellular MTs are dynamic, a quality essential for their role in cell physiological activities. During interphase, MTs serve as tracks for motor‐driven transport of organelles and vesicles, characterized by low catastrophe and growth frequencies.^7^ In contrast, spindle MTs experience a nearly tenfold increase in catastrophe rates and a reduction in the half‐time of MT turnover during mitosis.^8^ MT plus‐ and minus‐ends' dynamics are tightly controlled to ensure proper spindle assembly. This modulation of MT dynamics involves various MT‐binding proteins.^7^, ^9^, ^10^, ^11^

Liquid–liquid phase separation (LLPS) is a low‐affinity interaction primarily driven by hydrophobic interactions between molecules.^12^ Numerous membrane‐less organelles, such as P‐bodies, cell junction complex and Cajal bodies, form through phase separation.^13^, ^14^, ^15^ The intrinsically disordered region (IDR) was essential to drive the phase separation. LLPS proteins formed condensates which exhibit liquid‐like properties which recruit the binding proteins to have higher concentration than the macromolecules.^16^, ^17^ Recently, several MT‐binding proteins have been reported to have LLPS characteristics. LLPS, driven by the concentration of biomolecular condensates at specific times and locations, finely regulates the binding to MTs, enhancing efficiency in influencing MT dynamics. During mitosis, key regulators of MTs biomolecular condensates are discovered at the mitotic spindle matrix (BuGZ), MT plus‐ends, centrosomes (SPD5),^18^ spindle poles (NuMA),^19^, ^20^ inner centromere (INCENP/borealin and survivin),^21^ and MT branching nucleation (TPX2).^22^ Tau, a well‐studied MT‐binding protein implicated in various neurodegenerative diseases, undergoes LLPS.^23^ However, the role of aggregated tau in spindle assembly remains unclear. Here, we explore the latest advancements in LLPS MT‐binding proteins during mitosis, including MT plus‐ends tracking proteins such as EB1,^24^, ^25^ NuMA,^19^, ^20^ TPX2,^26^ BuGZ,^27^ and SKAP.^28^ Additionally, we discussed the potential role of Aurora‐A/B in coordinately mediated the phase separation of LLPS MT‐binding proteins during mitosis.

Through this review, we aim at the detailed mechanisms of MT‐binding proteins undergoing LLPS in vitro and in vivo, emphasizing the roles in orchestrating MT dynamics, spindle assembly and spindle orientation under physiological conditions. Furthermore, we propose potential directions for future research. Understanding the LLPS properties of MT‐binding proteins is pivotal for uncovering the mechanisms governing the cell cycle, one of the most critical cellular processes.

## 2 LLPS OF MT PLUS‐END BINDING PROTEINS

Around the plus tip of MTs, a complex network of proteins accumulates to manipulate the dynamics of the MT ends. These proteins spatially and temporally organize to regulate the physiological function of MTs, which is essential for the spindle formation, particularly.^29^, ^30^ Recent studies have highlighted the crucial role of MT‐binding proteins LLPS in organizing MT plus‐end dynamics.

**FIGURE 1:**
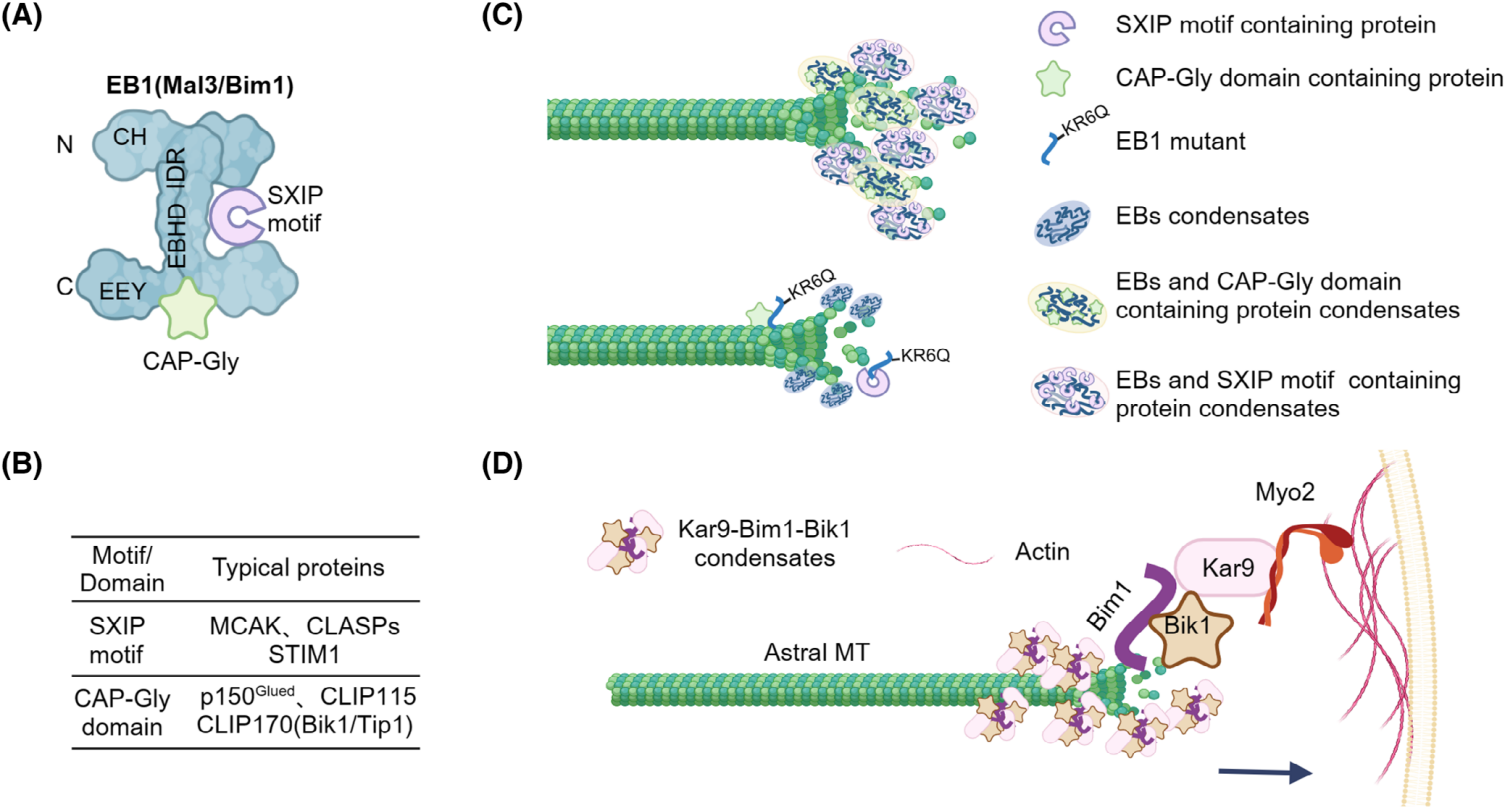
Phase separation of microtubule plus‐end binding proteins. (A) Domain organization of EB1 and its homologues. The N‐terminal comprises the calponin homology (CH) domain for microtubule (MT) binding, followed by the flexible linker and the homodimerization/heterodimerization domain (EBHD) responsible for dimerization. The C‐terminal features the Glu‐Glu‐Tyr (EEY) motif. EB1 forms homodimer through its EBHD. EB1 binds to the Cytoskeleton‐Associated Protein Glycine‐rich (CAP‐Gly) domain‐containing proteins according to the EEY motif. While EB1 interact with the proteins containing the Ser‐x‐Ile‐Pro (SXIP) motif by its EBHD. (B) Proteins containing the SXIP motif include mitotic centromere‐associated kinesin (MCAK), cytoplasmic linker protein (CLIP)‐associated proteins (CLASPs) and plus‐end tracking protein 150 (TIP150) bind to the EBHD of EB1 dimer, while those proteins with the CAP‐Gly domain contain p150^Glued^ of the dynactin complex, CLIP170 and CLIP115. (C) EBs undergo liquid–liquid phase separation (LLPS) and form co‐condensates with interacting proteins at the MT‐plus end. EB1 and EB3 undergo LLPS and create co‐condensates that enrich tubulin, enhancing MT growth rates. EB1 form condensates with the SxIP motif‐containing proteins, including TIP150 and MCAK. In budding yeast, Mal3 (EB1 homologue) forms co‐condensate with kinesin Tea2 and cargo Tip1(CLIP170 homologue, CAP‐Gly domain‐containing protein). The EB1‐KR6Q mutant which changed the two types positively charged residues to glutamine, fails to induce LLPS but binds to SxIP motif or CAP‐Gly domain‐containing proteins. However, without the EB1 LLPS, the SxIP motif‐containing proteins display the dispersed phase. (D) In vivo, Bim1(EB1 homologue), Bik1(CLIP170 homologue) and Kar9 (APC functional homologue) are the core of the microtubule plus‐end tracking proteins. They interacted with each other by multivalent interactions. Kar9‐Bim1‐Bik1 phase separates into condensates that track plus‐end of astral MTs. As Kar9 interacts with actin‐directed motor protein Myo2, which pulls the astral MTs to the actin filaments at the cortex. These droplets are essential for spindle positioning by tethering MTs to actin and generating the pulling force.

The MT end‐binding (EB) protein family, which includes EB1, EB2, and EB3, serves as pivotal and scaffold proteins responsible for tracking the plus‐ends of MTs.^31^ These EBs autonomously track the growing MT plus‐ends and stimulate their elongation, operating independently of other proteins in vitro. Notably, EBs are evolutionarily conserved, the N‐terminal calponin homology (CH) domain of EB1 plays a central role in MT binding, recognizing the GTP‐/GDP‐Pi capped tubulin state.^32^ The binding region of EB1 extends to the dynamic MT tips, forming MT comets. A flexible linker and subsequent coiled‐coil domain, along with the homodimerization/heterodimerization domain (EBHD), are responsible for dimerization.^33^ The C‐terminal region of EB1 contains a conserved Glu‐Glu‐Tyr (EEY) motif (Figure 1A).^34^

EB1 plays a crucial role in recruiting various plus‐end tracking proteins to MT plus‐ends through its EBHD or EEY motif.^35^ EBs, dimerize through the EBHD domain, forming two identical interfaces within the coiled‐coil four‐helix bundle. This dimerization facilitates the recruitment of proteins containing Ser‐x‐Ile‐Pro (SXIP) motifs or LxxPTPh motifs.^32^ Notable examples of such recruited proteins include mitotic centromere‐associated kinesin (MCAK) in Hela cells,^36^ cytoplasmic linker protein (CLIP)‐associated proteins (CLASPs) in CHO‐K1 and COS‐7 cells,^37^ and TIP150 in Hela cells and 293T cells.^38^ The conserved EEY motif in EB1, bearing similarity to the C‐terminal of α‐tubulin, plays a crucial role in binding to Cytoskeleton‐Associated Protein Glycine‐rich (CAP‐Gly) proteins.^39^, ^40^ This interaction includes proteins such as p150^Glued^ of the dynactin complex in HCT116 cells, MDCK cells, and U2OS, in CHO‐K1 and COS‐7 cells,^41^ CLIP170 in CHO‐K1, COS‐1 cells and COS‐7 cells^42^ and CLIP115 in COS‐1 cells^43^ (Figure 1B). The involvement of EB1 has been demonstrated to exert a significant influence on MT dynamics. In vitro, 7 μM free Alexa488‐labelled tubulin with 400 nM dimeric EB1 highly increased the catastrophe frequency from 0.10 ± 0.03 to 3.56 ± 0.51 min^−1^ on the pre‐existed tetra‐rhodamine‐labelled, GMPCPP‐stabilized seeds. Even 200 nM EB1 can increase the catastrophe frequency from 0.14 ± 0.02 to 1.07 ± 0.07 min^−1^ in presence of 8 μM free Alexa 488‐labelled tubulin.^44^ Thus EB1 strongly increases catastrophe frequency in vitro. However, in vivo, EB1 promotes persistent MT growth by suppressing catastrophe activity. This dual role suggests that proteins in vivo interacting with EB1 play a vital role in the precise regulation of MT dynamics.

In budding yeast, Mal3 (EB1 homologue), Kar9 and CLIP170 all exhibit LLPS properties.^24^, ^25^ The EB1 comets dynamically fuse into biomolecular condensates in living cells, recovering to 60% of their original fluorescence intensity within 1 min after photobleaching. Real‐time imaging shows that smaller EB1 condensates fuse with others to form larger ones. In vitro, the recombinant EB1‐GFP protein undergoes LLPS when the concentration exceeds 4.88 μM. The EB1 droplets enrich the tubulin concentration and form co‐condensates with the interacting proteins, including TIP150 or MACK (Figure 1B).^36^, ^45^

The IDR in the linker region of EB1, is not sufficient for the LLPS in living cells. Under physiological conditions, the integrity of EB1 is essential for the formation of condensates. Different regions of EB1 interact with each other, as demonstrated by Song et al. using a nuclear magnetic resonance (NMR) chemical shift perturbation assay. The IDR region interacts with the CH, EBHD and the C‐terminal tail, while the C‐terminal tail interacts with EBHD. Two types of positively charged residues, lysine and arginine (a total of six), in IDR region are crucial for the EB1 phase separation. The EB1‐KR6Q mutant fails to drive the phase separation but interacts normally with proteins containing SXIP motifs (CLASP1/2, TIP150 and MACK) or CAP‐Gly domains (p150^Glued^) (Figure 1C).^24^, ^36^, ^45^

Although some IDRs are enough to drive phase separation, each IDR seems unique to the protein in forming phase separation, and not always interchangeable between proteins. For instance, substituting hnRNPA1 IDR for EB1 IDR (referred to as EB1‐hnRNPA1 chimera) enhances phase separation ability and restores plus‐end tracking activity. However, other chimeras, such as EB1‐FUS and EB1‐TDP43, fail to form condensates and cannot restore plus‐end tracking activity.^24^ The reason could be the 3D structure of each molecule is different and therefore requires IDR in certain length and plasticity in integrating with the rest of the molecule when forming condensation. Cryo‐electron microscopy (Cryo‐EM) and AI technique could be a powerful tool in predicting the motif or structure necessary for the phase separation. Actually, IDRs contain two types of sequences that drive the phase separation, including the low‐complexity region (LCR) and short linear motifs (SLiM). On one hand, IDR proteins frequently harbour LCRs that increase the non‐stoichiometric multivalent interactions. On the other hand, the disordered SLiMs are always recognized as molecular recognition features that ≤8 amino acid residues in length. SLiMs interact with the folded domains that are mediated by hydrophobic contacts, electrostatic interactions and so on. The interaction influences the specificity of LLPS condensates.^46^, ^47^ Such as the adaptor protein Nck has a 50‐residues linker that contains two linear motifs, which increases the driving force for the phase‐separation of Nck.^47^, ^48^

In yeast, the role of IDRs in initiating LLPS is well explained by examples of studies on EB1. Mal3 (The EB1 homologue in *Schizosaccharomyces pombe*), forms a complex with Tea2 (kinesin‐7 homologue) and Tip1 (CLIP170 homologue) at the MT plus ends.^49^, ^50^ Tea2 and Tip1 localization depends on Mal3 in vivo and in vitro. In crowding conditions with PEG‐35k, purified Mal3, Tea2 and Tip1 undergo phase separation independently of MTs.^51^ The motor Tea2 transports the condensate towards the plus‐ends. Cryo‐electron tomography (Cryo‐ET) analysis of MT ends shows an obvious end‐decorated coat at growing MTs when Mal3, Tea2 and Tip1 are added, but not with Mal3 alone. Non‐stoichiometric Tip1 and Mal3 accumulate at MT plus‐ends as Tea2 concentration increases, indicating that Mal3‐Tea2‐Tip1 condensates are not limited by one‐on‐one interactions.^51^ The property is similar to Kar9 in *Saccharomyces cerevisiae*. Unlike human‐EB1, Mal3 possesses two IDRs, IDR1 between the CH domain and EB homology domain (EBHD), and the C‐terminal IDR2. Multivalency induces phase separation of Mal3‐Tea2‐Tip1 at plus‐ends. IDR1 is essential for comet formation on MT ends, while IDR2 contributes to interaction with the MT lattices. Both IDR1 and IDR2 are required for Mal3 self‐interaction and interaction with motors. IDR1 and EBHD are essential for recruiting Tip1, and IDR2 enhances motor‐dependent accumulation of Tip1 at growing ends. The detailed interaction domains and theoretical models are clearly outlined.^51^ However, whether Mal3 forms condensates or co‐condensates with Tea2 and Tip1 under physiological conditions remains unknown, requiring further studies to unveil the role of Mal3 phase separation in spindle assembly and mitosis.

In human cells, CLIP170 undergoes LLPS at MT plus‐ends. Recombinant CLIP170 and EB3 form droplets independently. Miesch et al. reveal that EB3 co‐condenses with CLIP170, increasing the amount of both proteins in the dense phase. In vitro, EB1‐CLIP170 co‐condensates enrich tubulin at growing MT tips, facilitating MT growth rates (Figure 1B).^25^

Bim1 (the homologue of EB1 in *S. cerevisiae*) forms condensates with Kar9 (functional homologue of APC, MACF, and SLAIN) and Bik1 (orthologue of CLIP‐170).^52^, ^53^ Kar9 is located at individual MT plus‐ends to perform specific functions as an MT‐actin crosslinking factor, tethers the MT plus‐end to the actin filaments at the plasma membrane or chromosomes.^54^, ^55^ Kar9 localization to the plus‐end of MT depends on Bim1. Kar9 interacts with Bik1 and the actin‐directed motor protein Myo2, pulling the MT to the actin filaments at the cortex. Kar9, Bim1 and Bik1 mutually interact (Figure 1D). The N‐terminal of Kar9 is essential for self‐association and binding with Myo2.^56^ The C‐terminal of Kar9, containing SXIP and LxxPTPh motifs, interacts with the EBHD of Bim1. Similar to EB1, the CH domain of Bim1 is responsible for MT‐binding and self‐interaction, while the C‐terminal tail binds to CAP‐Gly domain‐containing proteins like Bik1. In vitro, Kar9‐Bim1‐Bik1 phase separates into condensates independently of MT.^52^ Kar9 promotes phase separation of Bim1 and Bik1 over a broad range of sodium chloride and protein concentration. Only the full‐length Kar9, along with Bim1, drives phase separation. Kar9 N‐terminal domain dimer forms three interfaces that regulate self‐association. However, the self‐associated Kar9 undergoes phase separation only in the presence of Bim1. Notably, the IDR of Kar9 is not sufficient to drive phase separation.^52^

Plus‐end tracking proteins have been demonstrated to form condensates through multivalency interactions in various organisms. Understanding the physiological functions of these condensates is of primary concern. First, as MT‐binding proteins, it is essential to measure whether phase separation mediates MT dynamics. The LLPS property of EB1/Mal3/Kar9 is crucial for tip‐tracking activity. EB1 KR6Q mutant deficient in LLPS is unable to mediate MT plus‐end dynamics.^24^ Additionally, EB3 is another member of EB family, which is the core component of the plus‐end complex. Both EB1 and EB3 have high ability to regulate the plus‐end tracking proteins and also control the organization of MT minus ends by attaching the MTs to Golgi membranes.^57^ The two proteins have many common interactors, such as CLIP170 and CLASPs. Similar to EB1, EB3/CLIP170 co‐condensates with tubulin to enhance MT growth (Figure 1). Interestingly, different cells seem to have a preference in expressing EB3 or EB1 to regulate MT. EB3 was highly regulated during myogenic differentiation, whereas the EB1 transcription and translation levels remained unchanged.^57^ However, the expression pattern of EB1 and EB3 is somehow different in neurons. EB3 is mainly expressed in the central nervous system that links MTs and actin, whereas EB1 was implicated in axonal transport.^58^, ^59^, ^60^ Therefore, EB1 and EB3 may not have a simple antagonistic or synergistic relation. Similar to EB1, EB3/CLIP170 co‐condensates with tubulin to enhance MT growth (Figure 1).

The cellular functions of MT plus‐end complex LLPS are further analysed. The EB1 KR6Q mutant, unable to undergo LLPS but still recruiting interacting proteins, fails to form bipolar spindles and achieve accurate chromosome segregation, exhibiting spindle position defects.^24^ These findings indicate that EB1 LLPS is essential for spindle positioning and kinetochore‐MT attachments. In vivo, Kar9‐Bim1‐Bik1 condensates on astral MT ends. The phase separation of Kar9 enables tracking of both growing and shrinking MT plus‐ends, contributing to Kar9's role in tethering MTs to actin filaments in the cortex (Figure 1D). Consistent tracking facilitates pulling forces and supports spindle positioning.

LLPS is formed through multiple dynamic and weak interactions in non‐stoichiometric accumulation. These condensates efficiently perform their biological functions and can quickly transform from one complex to another. Various proteins track growing tips dependent on EB1, LLPS provides insights into how EB1 coordinates binding to different proteins spatially and temporally. Investigating the role of phase‐separated MT plus‐end in vivo holds promise for a deeper understanding of their cellular functions.

## 3 LLPS IN REGULATING kinetochore‐MTs ATTACHMENT

**FIGURE 2:**
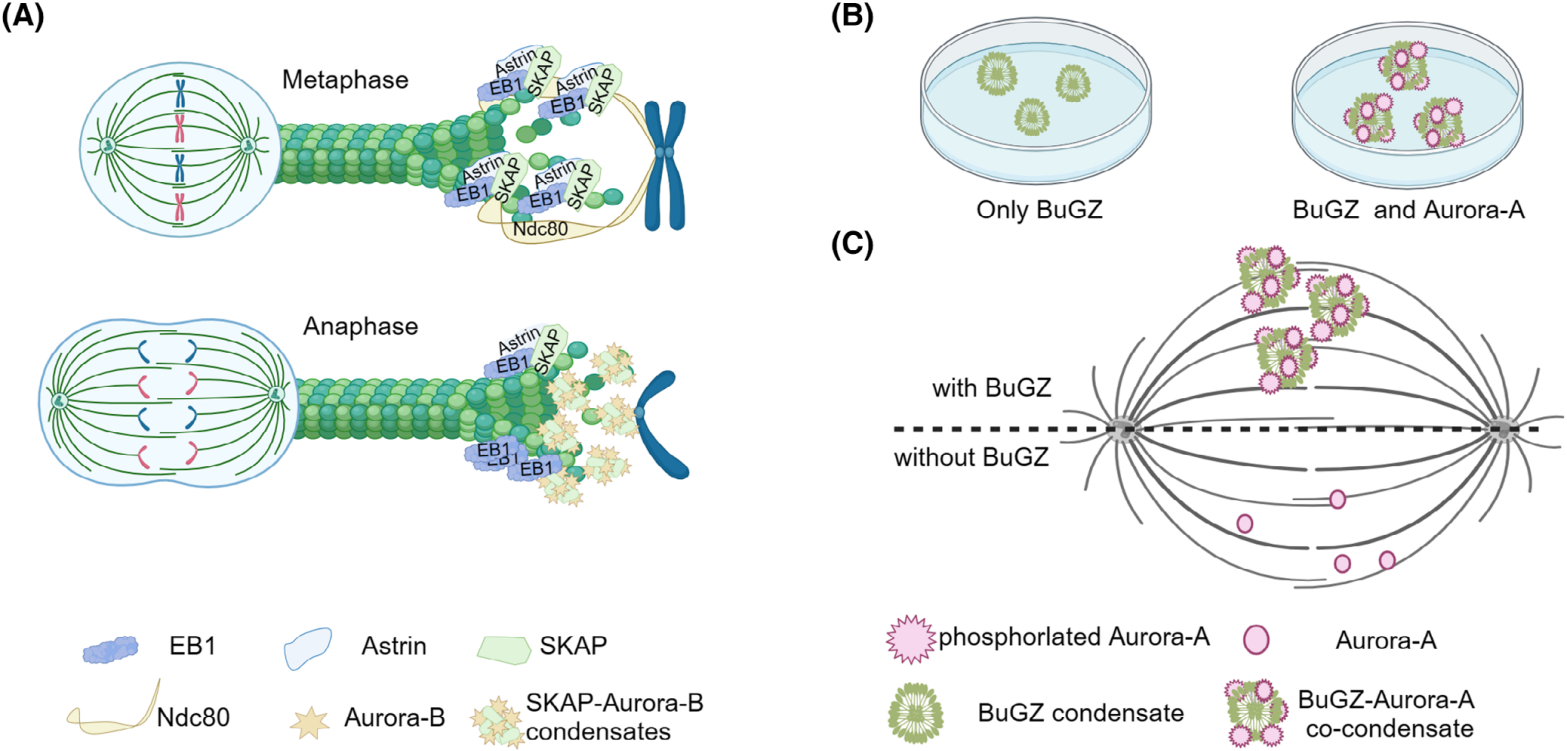
Phase separation of microtubule‐binding proteins during microtubule‐kinetochore attachment. (A) SKAP's phase separation governing kinetochore‐MT attachment. During the metaphase, SKAP interacts with EB1 and tubulin to track the plus end of spindle MTs. The astrin‐SKAP complex forms an integrated interface with NDC80, which is crucial for tethering MTs to the intra‐kinetochore. During the metaphase‐to‐anaphase transition, SKAP forms droplets with Aurora‐B. SKAP‐Aurora‐B co‐condensates guide the end‐on capture of dynamic kinetochore‐microtubules (MTs). (B) In vitro phase separation of BuGZ. Without the presence of tubulin and MT, BuGZ undergoes phase separation in vitro. BuGZ binds to the kinase domain of Aurora‐A and promotes the auto‐phosphorylation of Aurora‐A. The activated Aurora‐A forms co‐condensates with BuGZ. (C) With BuGZ, BuGZ and Aurora‐A form co‐condensates on spindle MTs, which facilitating MT bundling and enriching tubulin. High concentration of tubulin dimers stimulates MT polymerization. However, without BuGZ, the phosphorylated Aurora‐A decreased on spindle MTs. The decreased BuGZ and Aurora‐A lead to prolonged prometaphase arrest and cell death.

SKAP forms a complex with astrin, localizing to spindle poles and kinetochores during mitosis, which is pivotal for the transition to anaphase.^61^ SKAP tracks the plus‐ends of MTs, and SKAP's interaction with EB1 and binding to α/β‐tubulin regions play crucial roles in plus‐end tracking.^62^ SKAP binds to tubulin in solution, enhancing tubulin polymerization, and the plus‐end tracking ability of SKAP is essential for astral MTs to attach to the cell cortex for spindle positioning. SKAP interacts with MIS13, a subunit of the Mis12 complex in the NDC80 complex, confining its kinetochore localization.^63^ The astrin‐SKAP complex synergizes with the NDC80 complex, forming an integrated interface, providing intra‐kinetochore tethering and stabilizing MT interactions (Figure 2A).^64^ Unlike other complexes, astrin‐SKAP is recruited to the end‐on sides of kinetochores, and SKAP deletion impairs spindle pole integrity and destabilizes kinetochore fibres, leading to abnormal spindle formation.^62^, ^65^ Kinetochores lacking SKAP exhibit slower movement on both polymerizing and depolymerizing MTs, indicating SKAP's role in increasing the force for rescuing MTs and decreasing friction.^61^

Recent work by Zhang et al. highlights the formation of a complex between SKAP and Aurora‐B through phase separation, a process dependent on the C‐terminal region of SKAP. In vitro experiments demonstrated that SKAP spontaneously forms high‐fluidity droplets, recruiting Aurora‐B into these structures. The study by Song et al. further elucidates the role of astrin‐SKAP in transitioning from mono‐oriented kinetochore attachments to end‐on attachments.^66^ This mechanism involves the protection of mono‐oriented attachments by sensing changes specific to end‐on capture and stabilizing these attachments. The phase‐separation ability of SKAP emerges as crucial for correcting mis‐attachments by compartmentalizing Aurora‐B and guiding the end‐on capture of dynamic kinetochore‐MTs (Figure 2A).^28^ However, the underlying mechanism leading to increased chromosome segregation errors in LLPS‐deficient SKAP mutants remains unknown. Potential factors, such as altered plus‐ends tracking on MTs or changes in Aurora‐B concentration, may contribute to the observed effects. These findings open avenues for further research into the intricate processes governing kinetochore‐MT attachment and the role of SKAP's LLPS.

## 4 LLPS IN THE ASSEMBLY OF THE SPINDLE MATRIX

BuGZ, a crucial component of the lamin‐B spindle matrix in Xenopus, plays a pivotal role in regulating spindle morphogenesis by mediating kinetochore attachment to Bub3, facilitating chromosome alignment.^67^ Bub3, a spindle assembly checkpoint (SAC) protein bound to kinetochores, generates signals for chromosome alignment. Despite its weak binding to spindle MTs, BuGZ contains an MT‐binding domain in its N‐terminus, crucial for the efficient loading of Bub3 onto kinetochores. BuGZ initiates MT attachment to kinetochores by directly binding and stabilizing Bub3, mediated by its conserved GLEBS motif.^67^ Additionally, the interaction between BuGZ and MT promotes spindle MT assembly, as its deletion leads to prolonged prometaphase arrest and cell death. Jiang et al. discovered that BuGZ undergoes MT‐independent phase separation with temperature‐sensitive features. Compared with the high concentration of BuGZ, low concentration of purified BuGZ form condensate at higher temperature. While the MT‐binding domain deleted BuGZ, named xBuGZ∆N, exhibits the similar ability to coacervate. The conserved phenylalanine (F) and tyrosine (Y) residues drive BuGZ self‐interaction within condensates (Figure 2B). Mutating these conserved Fs and Ys into serine (xBuGZ13S) increases the minimum concentration and temperature to undergo phase separation. In the presence of MT, BuGZ forms more droplets along MTs. These condensates bind to MTs, facilitating MT bundling, and enriching tubulin. The BuGZ droplets promoted high tubulin concentration and stimulated MT polymerization. The endogenous concentration of BuGZ was high enough to undergo phase separation during spindle assembly.^68^ The mutants xBuGZ13S and xBuGZ∆N failed to promote MT assembly from spindle matrix, indicating the phase separation and MT‐binding ability are indispensable for promoting tubulin concentration and spindle MT assembly.

Intriguingly, Aurora‐A auto‐phosphorylates and forms co‐condensates with BuGZ in vitro. BuGZ binds to the kinase domain of Aurora‐A through its two zinc fingers and promotes the activation of Aurora‐A. Aurora‐A fused into the BuGZ droplets in vitro (Figure 2B). Depletion of BuGZ decreases phosphorylated Aurora‐A on spindle MTs (Figure 2C).^27^, ^69^ The mutants xBuGZ13S and xBuGZ∆N failed to activate Aurora‐A during mitosis, indicating the role of BuGZ LLPS in regulating Aurora‐A. Furthermore, BuGZ reduces the phosphorylation of MCAK at S196 in *Xenopus* egg extracts, and then the inactivated MCAK increases the spindle MT assembly. These results suggest a broader regulatory impact of BuGZ LLPS on spindle assembly regulation.^27^

BuGZ's MT‐independent phase separation, coupled with its dual role in Bub3‐mediated kinetochore attachment and direct promotion of spindle assembly, underscores its significance in coordinating various processes critical for proper spindle matrix assembly and function.^68^ Further investigations are warranted to elucidate the detailed mechanisms underlying BuGZ's regulatory interactions during spindle assembly and its implications in cellular processes.

## 5 LLPS IN FACILITATING BRANCHING MT NUCLEATION

**FIGURE 3:**
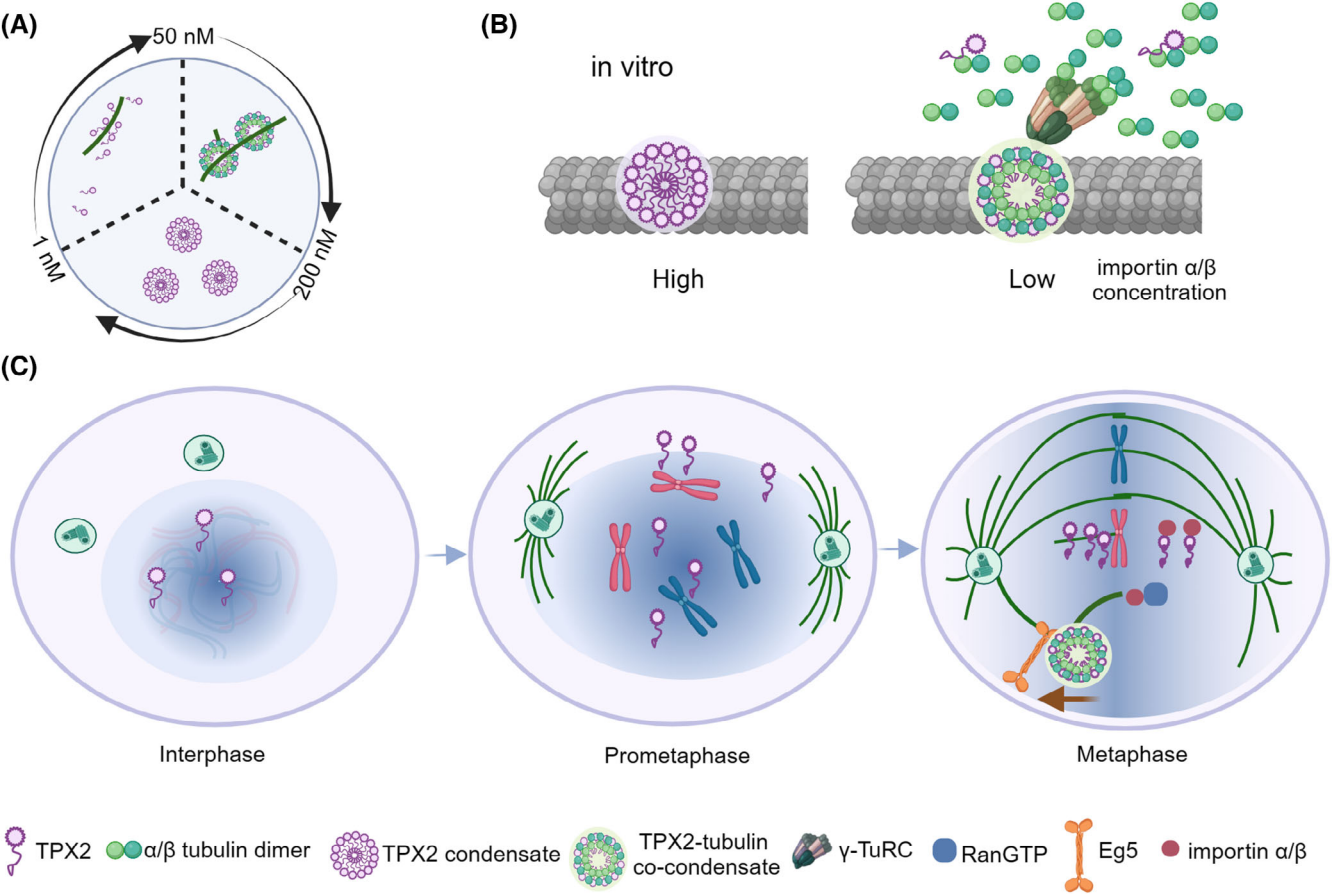
TPX2 phase separation with tubulin enhances branching MT nucleation efficiency. (A) TPX2 phase separation and co‐condensates with tubulin. At 1 nM concentration, TPX2 localized on MTs. As the concentration increased to 50 nM, TPX2 forms droplets with tubulin. The co‐condensation localized on MTs. Even without tubulin and MT, TPX2 undergoes phase separation after the concentration over 200 nM. (B) High Importin α/β concentration inhibit TPX2 condensation. With the low importin α/β concentration, TPX2‐tubulin co‐condensates located onto pre‐existing MTs and initiates branching MT nucleation. High importin α/β concentration inhibits the formation of TPX2 droplets and TPX2‐tubulin co‐condensates in vitro. (C) In Vivo TPX2 Activation and Efficiency of Branching MT Nucleation. In vivo, activated TPX2 concentration depends on RanGTP. RanGTP decreases from chromosomes to spindle poles, indicating high TPX2 concentration near chromosomes. During interphase, TPX2 concentration is low. After entering the prometaphase, RanGTP concentration increased and the active importin α/β reduced. During metaphase, TPX2‐tubulin co‐condensation occurs near chromosomes. TPX2‐tubulin droplets increase the efficiency of branching MT nucleation. However, near the spindle poles, as the RanGTP decreased, the active importin α/β binds to TPX2 to inhibit the formation of TPX2‐tubulin droplets. Other than branching MT nucleation, TPX2 concentration influences the spindle morphology through interacting with Eg5. The spindle MT localization of Eg5 is TPX2‐dependent; whether Eg5 can phase separate with TPX2 remains unknown.

The spindle assembly regulator, Targeting Protein for Xenopus Kinesin‐like protein 2 (TPX2), plays a multifaceted role in spindle assembly and orientation. Proper TPX2 levels are essential for spindle integrity, and its dysregulation results in aberrant spindle morphology.^70^, ^71^ TPX2 undergoes dynamic localization changes during the cell cycle, translocating from the nucleus to spindle poles and eventually to the midbody. TPX2's involvement in branching MT nucleation is particularly noteworthy. It interacts with tubulin, promoting MT formation and bundling.^72^ At different concentration ranges, TPX2 exhibit different status. In vitro studies reveal that TPX2 undergoes phase separation, forming droplets at 50 nM. Co‐condensation with tubulin occurs in the presence of TPX2, demonstrating its role in mediating branching MT nucleation. TPX2 phase separates at 200 nM without tubulin, while 50 nM TPX2 forms co‐condensates with tubulin. However, only 1 nM TPX2 localized to MTs (Figure 3A).^26^ In Xenopus meiotic cytosol, the physiologically relevant concentration of TPX2 (about 25–100 nM) forms co‐condensates with tubulin on pre‐existing MTs and mediates branching MT nucleation.^26^

The N‐terminal domain of TPX2 is responsible for tubulin recruitment and phase separation, while the C‐terminal domain is crucial for branching MT nucleation.^26^ The minimal active domains are the three α‐helical domains, including α5, α6 and α7, named TPX2^α5–α7^. TPX2^α5–α7^ is capable of binding to MTs and undergoes phase separation. Guo et al. identified the structure of TPX2^α5–α7^ on MT lattice using magic‐angle‐spinning NMR. TPX2^α5–α7^ is intrinsically disordered and adopts a folded structure to bind to MTs. The aromatic and hydrophobic residues of TPX2^α5–α7^ play a crucial role in regulating the binding to the MT lattice.^73^ The central region of TPX2 binds to the MT, αβ‐tubulin dimers, and the C‐terminal target Eg5 motors. NMR‐guided docking indicates that the direct interaction between γ‐TuRC and TPX2 is important in initiating MT nucleation. Other than the TPX2 and γ‐TuRC, augmin and Ran‐GTP are essential for the branching MT nucleation. These factors regulate this process spatially and temporally. TPX2 binds to the pre‐existing MT. A hydrodynamic Rayleigh‐Plateau instability drives TPX2 to form regularly spaced droplets on MTs. Higher TPX2 concentration increases the space between droplets. These droplets colocalized with augmin. With TPX2 and augmin, the ratio of γ‐TuRC on MTs to γ‐TuRC increased about 10‐fold. From these droplets, MT branches nucleated.^74^

Activation of TPX2 occurs upon binding with Ran‐GTP, a process regulated by importin α/β heterodimers.^72^, ^75^ Importin α/β concentration‐dependent reduction in TPX2‐tubulin co‐condensation is observed in vitro (Figure 3B).^26^, ^76^ In vivo, TPX2 stimulates MT‐dependent nucleation in collaboration with Ran‐GTP, enhancing branching MT nucleation efficiency.^77^ From chromosomes to spindle poles, the concentration of Ran‐GTP decreases while the active importin α/β increases. The condensation of TPX2‐tubulin occurs near chromosomes, facilitating MT nucleation, and sharply diminishes once the concentration of activated TPX2 falls below the threshold for phase separation (Figure 3C).^26^, ^76^ The formation of TPX2‐tubulin condensation significantly boosts the efficiency of branching MT nucleation by approximately 100‐fold. The phase separation property of TPX2‐tubulin enhances the rates and efficiency in physiological contexts.^26^

TPX2 concentration influences spindle architecture. The cells treated with TPX2 siRNA, decreased the recruitment of Eg5 to spindle poles and MT asters, and therefore modulated the spindle morphology. TPX2‐Eg5 interaction is crucial for kinetochore fibre formation and chromosome congression.^78^ Disrupting this interaction hampers the spindle MT localization of Eg5; however, the activity of Eg5 does not impact the spindle MT localization of TPX2. TPX2 also influences the velocity of Eg5‐dependent MT gliding and the accumulation of motor proteins on MTs (Figure 3C). It is worth noting that TPX2 itself undergoes phase separation at higher concentrations (about 200 nM), while forming co‐condensates with tubulin at lower concentration of 50 nM. Therefore, TPX2‐tubulin co‐condensates are for efficient branching MT nucleation. However, whether TPX2 co‐condensates with Eg5 to mediate its spindle localization and motility remains to be elucidated.

Collaboration between TPX2 and Aurora‐A is vital for connecting MTs with kinetochores during spindle formation.^79^ TPX2 activates Aurora‐A, and their interaction is characterized by a 43‐residue domain of TPX2 binding to two separate stretches on Aurora‐A. Even though the binding site of PP1 (the phosphatase of Aurora‐A) is distant from TPX2's binding site on Aurora‐A, TPX2 inhibits the dephosphorylation of Aurora‐A by protecting Thr288 from dephosphorylation and inducing conformational changes in the activation segment of Aurora‐A.^79^ In vitro, TPX2 increases the phosphorylation activity of Aurora‐A and prevents its dephosphorylation.^80^, ^81^ TPX2 depletion affects the binding affinity of Aurora‐A to spindle MTs, resulting in bipolar spindle defects.^82^ On the other hand, disrupting Aurora‐A affects the function but not the location of TPX2. The mechanism is still unclear. Like TPX2‐tubulin co‐condensates, we speculated that phase separated TPX2 may form co‐condensates with Aurora‐A, which protects Aurora‐A from dephosphorylation.

In summary, TPX2's phase separation properties play a pivotal role in branching MT nucleation, spindle morphology modulation, and its interactions with key regulatory proteins such as tubulin, Eg5 and Aurora‐A. Further investigations are needed to unravel the intricacies of TPX2‐mediated spindle assembly and its implications in cellular processes.

## 6 LLPS FACILITATES SPINDLE FORMATION AND ORIENTATION

Master regulators of MTs show the LLPS, which is proved to be necessary for accurate spindle formation and orientation. Nuclear and mitotic apparatus (NuMA) is a high molecular weight protein intricately involved in spindle organization during cell division.^83^ NuMA N‐terminus is the dynein‐dynactin binding motif. A self‐assembly coiled‐coil domain in the central which also contributes to the astral MTs capturing. The C‐terminal region holds a conserved 100‐amino acid MT‐binding motif, vital for stabilizing and bundling MTs. NuMA plays a pivotal role in anchoring dynein‐loaded MT plus‐end tips at cortical sites through interactions with Leucine‐glycine‐asparagine (LGN), Eg5, cortical 4.1R/G and the plasma membrane.^84^, ^85^

During interphase, NuMA predominantly localizes to the nucleus. As cells progress into mitosis, NuMA undergoes dynamic redistribution to the cell cortex and spindle poles.^83^ NuMA's interaction with LGN and Gαi subunits of heterotrimeric G proteins (Gαi) forms a complex crucial for spindle orientation. This complex recruits dynein to astral MTs, generating pulling forces on spindle poles, thereby determining spindle orientation.^86^, ^87^ The spindle pole localized NuMA recruit dynein to the minus ends of spindle MTs and clusters near the centrosomes along with dynein and dynactin.^88^ NuMA's spatial and temporal distribution, particularly at spindle poles, is critical for proper spindle positioning and pole formation.

**FIGURE 4:**
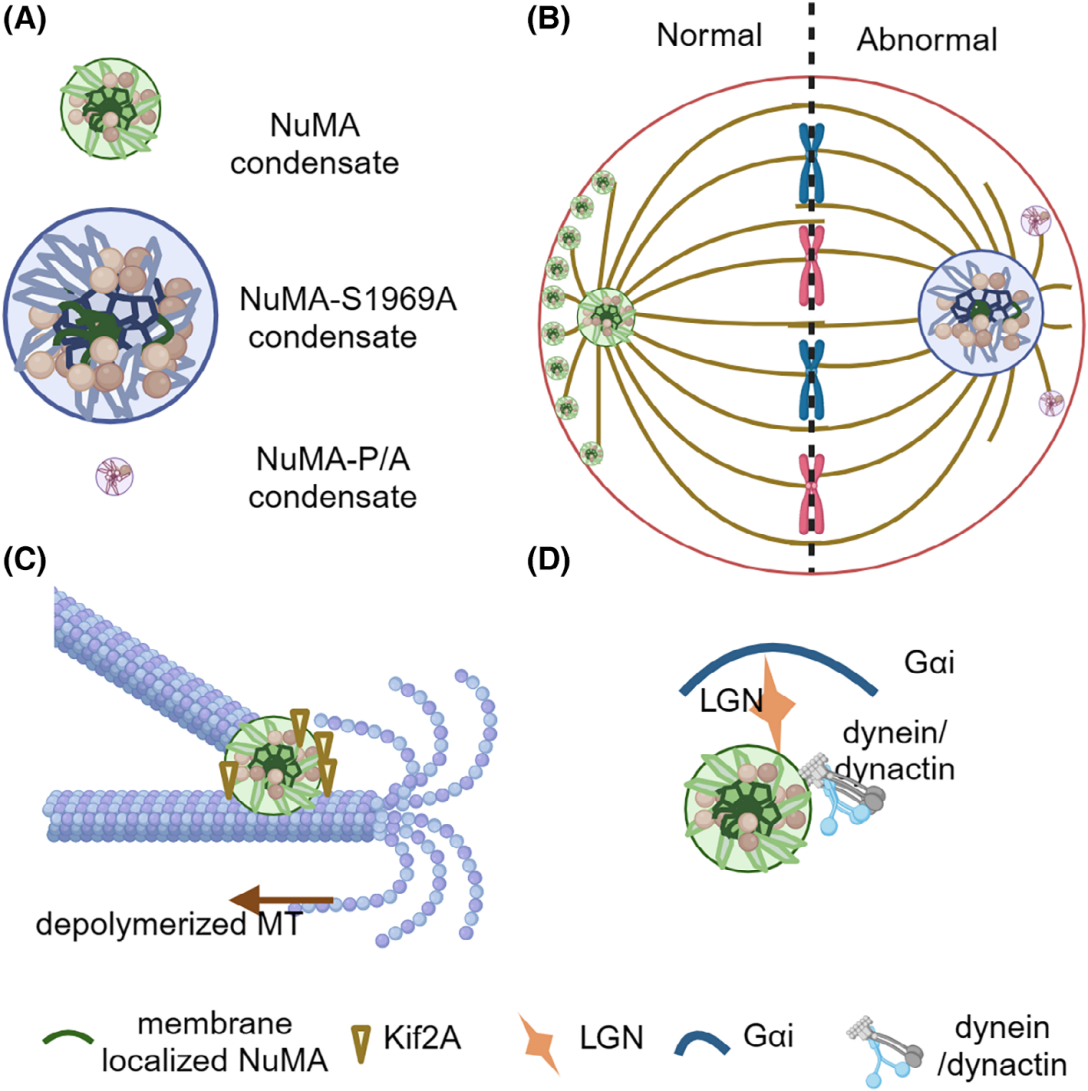
NuMA undergoes phase separation on spindle poles, regulating spindle formation. (A and B) NuMA droplet formation during G2/M transition. In vivo, during the G2/M transition, NuMA forms droplets on both spindle poles and the cell cortex. The NuMA condensates increase the tubulin concentration to promote the spindle pole assembly. The S1969A phosphorylation mutant, which could not be phosphorylated by Aurora‐A, forms larger droplets localized only to spindle poles. Another mutant, NuMA‐P/A (38 prolines mutated to alanines), forms smaller droplets than the C‐terminal wild‐type NuMA. The NuMA‐P/A mutation localized only to the cell cortex instead of spindle poles. (C) Recruitment of Kif2A to NuMA Droplets. NuMA interacts with Kif2A, an MT depolymerase. Kif2A is recruited into NuMA droplets on spindle poles, enhancing Kif2A concentration essential for spindle MT depolymerization on the minus‐ends. Kif2A droplet formation is dependent on the phase separation of NuMA. Kif2A depolymerizes spindle MTs and generates poleward MT flux. (D) NuMA droplets in cortical force generation. NuMA droplets assemble on the plasma membrane, interacting with MT motor dynein, dynactin, LGN and Gαi. The condensates enrich the interacting proteins and enhance the cortical force generation.

During G2/M transition, NuMA forms liquid droplets spontaneously and the C‐terminal NuMA forms droplets in vivo. This phase separation is observed in vitro through droplet formation and FRAP experiments. The C‐terminal NuMA concentrates tubulin in vitro, promoting spindle pole assembly by concentrating tubulins, binding to MTs, and recruiting essential regulators (Figure 4A).^19^, ^20^ Aurora‐A phosphorylation at S1969 regulates the phase separation, increasing the dynamics of NuMA droplets during mitosis. The S1969A mutant forms larger and unrecoverable droplets compared to the wild‐type NuMA (Figure 4A). In contrast to NuMA and NuMA‐S1969D, both localized to spindle poles and the cell cortex, NuMA‐S1969A was solely localized to the spindle poles in vivo (Figure 4B). The prolines in the C‐terminals contribute to the formation of droplets. Mutating the 38 prolines to alanines (NuMA‐P/A) decreases the diameter of droplets without reducing the MT‐binding ability (Figure 4A). Surprisingly, the location of the NuMA‐P/A mutation shifts from spindle poles to the cell cortex (Figure 4B).^20^ However, the mechanism behind the altered location remains elusive. Based on the localization patterns of these two mutant types and the characteristics of droplet size and fluidity, we speculate that the increased ability to form large or rigid droplets restricts the transport of NuMA from the centrosome to the cortex. The 1911–1925 amino acid motif of NuMA interacts with KifC1 to facilitate its localization to spindle poles.^19^ Kif2A was identified in NuMA droplets on spindle poles. Kif2A, an MT depolymerase, forms condensates dependent on NuMA on the spindle poles. The presence or absence of Kif2A has no influence on the spindle pole location of NuMA (Figure 4C). However, Kif2A's role in depolymerizing MTs is crucial for spindle length control. During mitosis, NuMA phase‐separated on the spindle poles is critical for recruiting Kif2A to depolymerize spindle MTs and generates poleward MT flux. Disrupting the phase separation leads to longer spindle length.^19^

Additionally, NuMA interacts with EB1 at the plus ends of astral MTs, contributing to their connection with the cell cortex.^89^ The NuMA condensates in the cell cortex enrich interacting proteins, including dynein, dynactin, LGN and Gαi (Figure 4D). While EB1 undergoes phase separation at MT plus‐ends, the co‐condensation of EB1 with NuMA requires further investigation. These findings highlight the intricate role of NuMA's phase separation in spindle pole formation, spindle orientation, and its interactions with key regulatory proteins such as Kif2A and EB1.

## 7 CELL CYCLE‐RELATED KINASES IN MODIFYING MT‐BINDING PROTEINS LLPS

Mitosis, a highly regulated process, involves the dynamic interplay of various kinases and phosphatases that modulate the behaviour of MT‐binding proteins, orchestrating the transition from interphase to M phase.^90^ Two conserved serine/threonine kinases, Aurora‐A and Aurora‐B, exhibit distinct subcellular localizations and contribute significantly to mitotic regulation.

Aurora‐A, localized at centrosomes during mitosis, ensures centrosome maturation and bipolar spindle establishment. Its co‐condensation with BuGZ droplets promotes Aurora‐A kinase activity on spindle MTs, regulating numerous MT‐binding proteins.^69^ Aurora‐A phosphorylates NuMA at three serine residues (Ser1969, Ser1991 and Ser2047), influencing spindle orientation without affecting NuMA's affinity for MTs or LGN.^91^ Notably, Aurora‐A phosphorylation of NuMA at S1969 reduces phase separation during mitosis. TPX2 enhances Aurora‐A activity, maintaining normal spindle length, and coordinating with Aurora‐A in regulating NuMA‐mediated spindle orientation. Overexpressed TPX2, potentially due to over‐aggregation, fails to recruit Aurora‐A, resulting in a super‐alignment phenotype.^92^ We hypothesize that the excessive aggregation of TPX2 reduces its ability to undergo phase separation, and the low mobility of TPX2 hinders the recruitment and activation of Aurora‐A. Additionally, this may also impact the phosphorylation of NuMA and its ability to form droplets. These findings indicate that Aurora‐A has the potential to coordinately regulate the phase separation of various proteins during mitosis.

Aurora‐B, located at centromeres and spindle mid‐zone, phosphorylates multiple MT‐binding elements at incorrectly attached kinetochores. In prometaphase, EB1 recruits Aurora‐B kinase to centromeres, allowing spindle MTs to regulate kinetochore phosphorylation.^93^ However, in human cells, EB1 interacts with Auroras but is not their substrate. EB1 inhibits the interaction between Aurora‐B and PP2A, preventing Aurora‐B dephosphorylation.^94^ Aurora‐B phosphorylates EB2, reducing its MT association, which is crucial for proper chromosome segregation.^95^ During different mitotic stages, Aurora‐A and Aurora‐B phosphorylate S176 of EB3, and this phosphorylation is enriched in the midbody, controlling cortical MT growth during mitotic exit.^96^ EB1 and EB3 undergo phase separation at the MT plus‐ends, whether Aurora‐A or Aurora‐B are fused to the droplets remains unknown. The condensates confine the proteins in the specific area and inhibit the protein diffused speed.

Furthermore, Aurora‐B phosphorylates outer kinetochore proteins, including the Ndc80 complex and the Ska complex, regulating kinetochore‐MT turnover and preventing attachment errors. This phosphorylation reduces MT binding while increasing MT catastrophe activity.^97^, ^98^ Interestingly, Aurora‐B's kinase activity at the kinetochore (rather than the centromere) is also dependent on Ska, especially in an MT‐dependent manner.^98^ Depletion of the Ska complex leads to the loss of Aurora‐B's phosphorylation function, impacting the subsequent phosphorylation of kinetochore substrates such as the Ndc80 complex and the inner kinetochore protein MCAK.^98^ The N‐terminal disordered loop region of Ska1 interacts with the C‐terminal region of the EB1 dimer, promoting the formation of an extended structure of the EB1‐Ska complex on the MT lattice.^99^ Disruption of the EB1‐Ska complex, through the depletion of EB1 or Ska, results in chromosomes scattering throughout the spindle. The absence of the EB1‐Ska complex leads to MT depolymerization and defective MT‐kinetochore attachment.^100^ The Ska complex, with its disordered loop region interacting with EB1, plays a key role in kinetochore‐MT attachment. While the Ska complex's role in phase separation is not explicitly reported, its involvement in the EB1‐Ska complex formation and Aurora‐B recruitment suggests the potential for phase separation at the kinetochore. These insights highlight the intricate regulatory roles of kinases such as Aurora‐A and Aurora‐B in modulating the phase separation dynamics of key MT‐binding proteins during mitosis, contributing to the precise orchestration of cellular events.

## 8 DISCUSSION AND FUTURE PERSPECTIVE

LLPS is a physical phenomenon that has been intensively studied for the last decades.^16^ Key events in cell activities have been proved to be mediated by LLPS on some proteins; for instance, MT‐binding proteins LLPS in the regulation of cytoskeleton dynamics.^12^, ^16^, ^17^, ^22^, ^26^ LLPS of MT‐binding proteins generates focal elevated concentration on the cytoskeleton and allows for important chemical reactions to occur swiftly, therefore, regulating the precise MT and actin filaments rearrangement. The utilization of an in vitro system provides a unique advantage over studying the phase separation of MT‐binding proteins and their contribution to MT array regulation.^24^, ^25^, ^26^, ^27^, ^68^, ^101^ The data from in vitro assay may provide a plausible explanation for the complicated dynamics of the MT array in vivo, such as spindle morphological changes in mitosis and meiosis. In this review, we summarize the LLPS of MT‐binding proteins and their roles in spindle MT regulation, for example, NuMA, EB1 and TPX2. From a larger perspective, more MT‐binding proteins may show similar LLPS under certain circumstances in manipulating MTs, such as katanin, kinesins and so on.

In the future, certain questions remain. First, the biological stepwise process of fusion between these droplets during the relatively short period of spindle formation and separation is elusive. For example, EB1 can form aggregates with different proteins, raising questions about whether these aggregates move together or disassemble into monomers before coalescing with other proteins. Second, the roles of potential factors that regulate the phase separation process, such as Aurora‐A, are enigmatic. Third, the droplets' size and fluidity control mechanisms in phase separation are worth the exploration. For example, the post‐translational modification especially phosphorylation is proved to regulate the LLPS size control process. Whether other post‐translational modifications that participate in centrosome maturation or spindle MT assembly also regulate LLPS are still unknown.^24^, ^28^, ^102^ As research progresses, addressing these questions will contribute to a more comprehensive understanding of the intricate processes involved in MT dynamics and organization. The in vitro MT system provides a more faithful representation of in vivo functionality, contributing to a comprehensive understanding of the roles these proteins play in MT organization and regulation. This approach lays the groundwork for future investigations into MT‐binding protein functions. Transmission electron microscope and immunoelectron microscopy are the most direct way to visualize the localization and formation of these condensations in vivo. With immune‐gold labelling, the precise subcellular location is more easily visible. Of note, the ability to form condensations and its biological functions is not an exact correspondence. More experiments from physiological conditions are essential to uncover the roles of MT‐binding proteins. In recent studies, the structure of TDP‐43 derived amyloid fibrils has been analysed by cryo‐EM, which is different from the previously described TDP‐43 structures in the number and relative orientation of the protofilaments including the fibril purified from patient tissue. TDP‐43 forms multiple fibril structures with different tertiary or quaternary structures in vivo, which all display the phase‐separation ability in vitro by the laser confocal fluorescence microscope or electron microscopy.^103^ That is to say, these in vitro‐formed condensates do not necessarily perform the same function. Hence, the cryo‐EM is a helpful tool to investigate whether the structures of the phase‐separated droplets from in vitro and physiological conditions are coincident or not. Additionally, more in vivo experimental results are needed to confirm the role of LLPS in the physiological state.

Our review mainly addresses the role of MT‐binding protein phase separation in the spindle assembly and orientation during mitosis. Actually, in mammalian oocytes, phase separation of MT regulatory factors is important for the acentrosomal spindle assembly during meiosis. The liquid‐like meiotic spindle domain (LISD) was identified and permeated the spindle poles. The LISD concentrates many MT regulatory factors including centrosomal proteins, MT minus‐end binding proteins and proteins that control MT nucleation and stability. The LISD assembly is dependent on Aurora‐A, TACC3 and CHC17. The LISD is essential for MT assembly and the stable acentrosomal spindles in meiotic spindle assembly.^104^, ^105^ In conclusion, phase separation is a widely existing principle to promote spindle assembly in different cells. We provide insights for further studies on the mechanisms underlying the LLPS of MT‐binding proteins and the relationship with its physiology function.

## AUTHOR CONTRIBUTIONS

SS wrote the manuscript and drew the figures. PL and JZ conceived the study and edited the manuscript. YY conceived the study and created the images. [Correction added on 22 July 2024, after first online publication: Author Contributions has been updated].

## CONFLICT OF INTEREST STATEMENT

The authors declare no conflicts of interest.
